# Helicases and human diseases

**DOI:** 10.3389/fgene.2015.00039

**Published:** 2015-02-12

**Authors:** Fumiaki Uchiumi, Masayuki Seki, Yasuhiro Furuichi

**Affiliations:** ^1^Department of Gene Regulation, Faculty of Pharmaceutical Sciences, Tokyo University of ScienceNoda, Japan; ^2^Department of Biochemistry, Faculty of Pharmaceutical Sciences, Tohoku Pharmaceutical UniversitySendai, Japan; ^3^GeneCare Research Institute Co., Ltd.Kamakura, Japan

**Keywords:** helicasees, genetic diseases, RecQ helicases, *Fanconi Anemia*, premature aging, cancer, RNA helicases

Recent progress in pharmaceutical sciences has made it possible for us to live longer and longer. For example, antibiotics and vaccines have been developed that were successfully administered to patients with infectious diseases. A number of effective drugs for specific diseases could be purified from natural resources or created by chemical synthesis, and recent recombinant DNA technologies have brought about antibody-drugs. It seems increasingly possible that a treatment for every disease could be established in the near future. Nevertheless, prevention or remedies for inherited age-related diseases, including cancer, have not yet been completely established. However, recent progresses in human genetics and molecular biology revealed that premature aging is caused by mutations on DNA helicase encoding genes (Bernstein et al., 2010). These exciting findings have encouraged scientists to research mechanisms of the age-related diseases.

DNA/RNA helicases are enzymes that unwind DNA/DNA, DNA/RNA, and RNA/RNA duplexes to execute and regulate DNA replication, recombination, repair, and transcription (Patel and Donmez, 2006). To date, numerous genes have been identified to encode helicases. Importantly, genetic studies have revealed that mutations in some of these genes are associated with certain human diseases, including *Xeroderma Pigmentosum* (XP), *Cockayne Syndrome* (CS), and *Werner Syndrome* (WS) (Puzianowska-Kuznicka and Kuznicki, 2005). Given that helicases play an important role in the regulation and maintenance of chromosomal DNAs, it might not be so difficult to understand that their dysfunction leads to unfavorable states. Nuclear events, such as nucleotide excision repair (NER), transcription coupled repair (TCR), and telomere maintenance, are thought to be individually affected by XPB/XPD, CSA/CSB and WRN helicases, respectively (Table 1). Because epigenetic changes and disruption of chromosomal integrity have been strongly suggested to correlate with cellular senescence, these helicases may be important factors to regulate aging and age-related diseases.

**Table 1:** **Helicases that associate with human diseases**.

**Helicase (*GENE ID*)**	**Disease**	**References**
BLM *(BLM*)	BS^a^^,^^b^	Ellis et al., 1995
CSA (*ERCC8*), CSB (*ERCC6*)	CS^a^^,^^d^	Henning et al., 1995
DDX11 (*DDX11*)	Warsaw breakage syndrome^d^	van der Lelij et al., 2010
FANCJ (*BRIP1*)	FA^b^^,^^c^	Levitus et al., 2005
IGHMBP2 (*IGHMBP2*)	SMARD1^d^, CMT2^d^	Grohmann et al., 2001; Cottenie et al., 2014
IFIH1 (*IFIH1*)	SLE^e^	Robinson et al., 2011
MCM4 (*MCM4*)	NKGCD, cancer	Hughes et al., 2012; Jackson et al., 2014
RECQ1/RECQL1 (*RECQL*)	Cancer	Sharma and Brosh, 2008
RECQL4 (*RECQL4*)	RTS^a^^,^^b^	Kitao et al., 1999
RTEL1 (*RTEL1*)	HHS^b^^,^^c^^,^^f^	Ballew et al., 2013
SETX (*SETX*)	ALS4^d^	Chen et al., 2004
TWINKLE (*c10orf2*)	MDS7^d^	Spelbrink et al., 2001
WRN (*WRN*)	WS^a^^,^^b^^,^^f^	Oshima et al., 1996
XPB (*ERCC3*), XPD (*ERCC2*)	XP^b^, CS^a^^,^^d^	Sung et al., 1993; Hwang et al., 1996

a Premature aging.

b Cancer or risk of cancer.

c Bone marrow failure.

d Impaired development of nervous system or deficiencies in neuromuscular junctions.

e Autoimmune disease.

f Telomere shortening.

ALS, amyotrophic lateral sclerosis; BS, Bloom syndrome; CMT, Charcot-Marie-Tooth disease; CS, Cockayne syndrome; FA, Fanconi anemia; HHS, Hoyeraal Hreidarsson syndrome (Dyskeratosis congenita); MDS, Mitochondrial DNA depletion syndrome; NKGCD, Natural killer cell and glucocorticoid deficiency with DNA repair defect; SLE, systemic lupus erythematosus; RTS, Rothmund-Thomson syndrome; SMARD1, spinal muscular atrophy with respiratory distress type 1; WS, Werner syndrome; XP, Xeroderma pigmentosum.

Despite great efforts being made to elucidate the properties of helicases on a molecular and cellular level, it seems that the gap from molecule to patient is still distant. In this research topic, authors have described and discussed the forefront of the helicase studies. It is very important to establish a molecular model of how helicases interact with DNA repair machinery. In the research topic, the properties of the FANCJ (BRIP1) that affect cancer and *Fanconi Anemia* (FA) development have been summarized (Brosh and Cantor, 2014). In order to assess the mechanisms of diseases, including cancer, which are caused by dysfunctions of helicases, several approaches could be applied. Genetic and expression analyses of samples from patients will enable us to discuss the alterations in both the quality of DNA and the quantity of RNA. Therefore, diagnosis/prognosis of cancer or age-related diseases will be possible by analyzing the *RECQ1* (*RECQL*) gene expression (Sharma, 2014). Based on the concept that helicases play important roles in the maintenance of chromosomal DNAs, novel therapeutics will be applicable for cancer therapy with siRNAs of the RECQL1 (RECQL) and WRN DNA helicase-encoding genes (Futami and Furuichi, 2015). The therapy is supported by experimental results showing that siRNA of the *RECQL* could be effectively applied for ovarian cancer treatment by inducing apoptosis (Matsushita et al., 2014). Structural analyses of the helicase protein molecules will provide their precise function in the process of DNA repair. The precise molecular structure models of the WRN and BLM helicases will contribute for a development of rational design of specific drugs to prevent aging and cancer (Kitano, 2014). Moreover, establishment of iPSCs from helicase deficient cells will contribute to the clinical tests to develop novel drugs that delay aging and age-related diseases (Shimamoto et al., 2015). Furthermore, studies on RNA helicases, especially those that are involved in immune responses, will contribute to developing strategies against viral infections. It was shown that DDX3 could be a novel therapeutic target for HIV-1 and HCV replication (Ariumi, 2014). Importantly, IFIH1, which controls anti-viral responses, will be a molecular target of diagnosis and treatment for systemic lupus erythematosus (SLE) (Oliveira et al., 2014). All these articles provide new insights into the molecular pathology of the helicase-associated diseases. Further studies on various helicases will not only contribute to diagnoses and treatment of specific diseases (Table 1) but also to prevention and next generation-therapeutics on cancer and age-related diseases.

## Conflict of interest statement

The authors declare that the research was conducted in the absence of any commercial or financial relationships that could be construed as a potential conflict of interest.
